# Supplementary material for: Are carbon emissions trading and green financial instruments synergistic? -Comprehensive quantitative research based on content analysis
Source: PLoS One. 2024 Mar 7;19(3):e0298601. doi: 10.1371/journal.pone.0298601 (PMC10919601; doi:10.1371/journal.pone.0298601)
Supplement: S1 Data — (ZIP) [file pone.0298601.s001.zip › Data/地级市Z_F工作报告.zip!/╕ú╜¿/─╧╞╜╩╨/└┤╫╘╣1⁄2╓┌║┼í╛┬φ┐╦╩2╛▌═°í┐_2016.docx]

各位代表：

现在，我代表南平市人民政府，向大会报告政府工作，请予审议，并请市政协各位委员和其他列席人员提出意见。

一、2015年和“十二五”时期经济社会发展回顾

2015年，在省委、省政府和市委的正确领导下，我市各级政府全面贯彻党的十八大和十八届三中、四中、五中全会精神，深入贯彻习近平总书记系列重要讲话和对福建工作的重要指示精神，按照“机制活、产业优、百姓富、生态美”要求，积极创建全国绿色发展示范区，发展效应显现。初步统计，全市生产总值1339.5亿元，增长9.1%；一般公共预算总收入124.3亿元、增长5.1%，地方一般公共预算收入86.4亿元、增长6.7%；全社会固定资产投资1802.5亿元，增长21.7%；外贸出口11.26亿美元、下降23.2%，实际利用外资1.45亿美元、增长21.1%；旅游接待人数2925万人次、收入371.6亿元，分别增长16.9%、18.9%；社会消费品零售总额504亿元，增长11.5%；居民消费价格总水平上涨1.6%；城镇居民人均可支配收入26120元、增长8.5%，农村居民人均可支配收入12265元、增长9%；城镇登记失业率3%；人口自然增长率6.86‰；年度节能减排任务全面完成。

一年来的主要工作和成效是：

（一）扩大有效需求，经济持续平稳增长。强化投资支撑，在建重点项目完成投资482.6亿元，合福高铁开通运营，京台高速公路南平段、延顺、邵光3条高速公路建成通车，华电邵武火电厂扩建等一批项目开工建设，向上争取财政资金139亿元。改进“5·13”投洽会，成功举办“11·16”茶博会，引进项目1466个、总投资3106亿元。增强消费拉动，拓宽武夷品牌产品销售渠道，新兴消费增长点加快培育。发挥政策效应，落实封闭转贷政策，累计为1005家企业提供68.2亿元贷款周转资金；出台做大国有融资担保、鼓励兼并重组、地产名优工业品目录、绿色产业发展基金等政策，有力支持实体经济发展。

（二）加快转型升级，产业发展提质增效。突出抓龙头铸链条建集群，新增5家企业“新三板”挂牌，新增入统规模以上工业企业162家、总数达到1109家；食品加工、机电制造、竹加工、新型轻纺、生物医药等产业产值占比提升1.8个百分点。太阳电缆、圣农等增资扩产项目投产，技改投资增长44.8%，列入省级智能制造重点项目20个。南平市省级高新技术产业园区上报待批，建瓯获批中国东南白酒名城，光泽获评全国食品工业强县，浦城列入全省首批创新驱动助力工程示范县。新认定中国驰名商标6件。建成一批电子商务集聚园区和交易平台，6个县（市）列入国家、省电子商务进农村示范县。加强三产业态整合，新增限额以上商贸企业、规模以上服务业企业209家，三产增加值增长12.3%，拉动GDP增长4.1个点。建阳建盏文化一条街等专业街区带动效应显现。新增国家3A级以上旅游景区11家，建成旅游集散中心6个、旅游厕所136座，武夷山列入国家公园体制试点，松溪获评全国休闲农业与乡村旅游示范县，旅游产品和服务设施进一步健全。

（三）夯实“三农”基础，现代农业加快发展。粮食生产保持稳定，农林牧渔业总产值增长4.8%。新评定市级农业产业化重点龙头企业128家，农业新技术示范推广158项。发展农民专业合作社4121家、家庭农场697家。流转耕地87.8万亩，建设高标准农田5.94万亩，新增丰产高效竹林30万亩。顺昌成立全省首家林业金融服务中心。新增“三品一标”认证26个、地理标志证明商标5个。解决8万农村人口饮水安全问题。完成撤渡建桥及危桥改造66座、农村公路安保工程326公里，新增13个乡镇通达三级以上干线公路，农村交通基础设施得到提升。

（四）共建美丽南平，城乡环境宜居宜业。深化户籍制度改革，农业转移人口市民化有序推进。新型城镇化建设步伐加快，光泽生态食品城试点、邵武产城融合试点、省级小城镇综合改革试点建设取得新进展。邵武列入国家新型城镇化综合试点、省级“多规合一”试点。“六个一”城建项目、614个宜居环境建设项目、16个“三边三节点”景观整治项目加快实施，拆除“两违”面积344万平方米。古村落、古建筑分级保护力度加大。持续实施“百千万”工程，142个美丽乡村加快建设，完成绿廊建设4.57万亩，“万人保洁”机制持续深化。

（五）建设新区新城，发展空间持续拓展。武夷新区建设全面提速，完成投资142.9亿元、增长38.3%。建阳顺利实现撤市设区，城市核心区、产业功能区和大交通网络布局进一步优化。高铁东站配套工程交付使用，新岭至高铁东站快速通道通车，4所学校竣工，云谷小区、歌剧院、艺术中心、轨道交通开工建设。延平新城高铁站前广场投入使用，从彦路一期、新港路一期、316国道炉下至东坑段改扩建工程动工建设。市老年人活动中心投入使用，南平新大桥拓宽改造工程建成通车，金山、大家厂体育公园主体工程建成，城市功能逐步完善。

（六）保护绿水青山，生态优势日益凸显。4个县（市）通过国家级生态县技术评估。造林绿化37.46万亩，水土流失综合治理42.1万亩。淘汰黄标车3890辆、燃煤锅炉290.3蒸吨，均超额完成省上下达任务。国家节能减排财政政策综合示范城市创建工作通过年度考核，国家循环经济等示范试点城市建设有序推进。严格执行“河长制”，主要河流水质全优。延平畜禽养殖污染第三方治理试点取得成效。农村环境综合整治和工业污染、大气环境、流域水环境、茶山等整治工作取得明显成效。

（七）突出精准扶贫，发展成果惠及民生。5个省级扶贫开发工作重点县落实帮扶项目271项、到位资金19.43亿元，“造福工程”危房改造5595户，3万人实现脱贫。政和创建全国扶贫小额信贷示范县。19项为民办实事项目全部完成，新增城镇就业2.3万人，农村劳动力转移就业5.3万人，保障性安居工程基本建成5060套。完成21所中小学扩容改造和公办幼儿园建设，全市中小学标准化建设通过验收，职业教育体系逐步构建，各类教育均衡协调发展。医药卫生体制综合改革试点全面实施，公立医院改革扎实推进，武夷总医院稳步发展。城乡居民社会养老保险“长缴多得”激励机制全面建立，城乡居民基本医疗保险实现政策一体化，城镇职工医疗保险基金实行市级统筹。成功承办全国首届青运会排球赛。人才队伍建设持续加强。妇女儿童、老龄和残疾人工作得到加强，社会福利和慈善事业稳步发展。村（居）委会换届选举工作顺利完成。安全生产标准化建设提升工程、道路交通安全综合整治加快推进，食品药品安全有效保障，社会安定稳定。民族宗教、外事侨务、气象防震、档案方志、老区库区等工作继续加强。军民融合深度发展，军政军民关系更加密切。

（八）践行“三严三实”，作风建设得到加强。深入开展“三严三实”专题教育，从严落实中央八项规定精神，持续整治“四风”，严格执行“约法三章”，全市“三公”经费下降21.57%。在全省率先公布“三张清单”，共取消、下放市级行政审批事项166项、公共服务事项75项。全面推进行政服务中心标准化建设，全面推广“一站式”服务。自觉接受人大、政协监督和社会监督，办理人大代表议案2件、建议144件，政协委员提案254件，满意和基本满意率分别为100%、99.6%。落实“三级联动、重心下移”工作机制，深化机关效能建设，强化“马上就办”，整治“庸懒散拖”。政府与法院、检察院、工会联系机制不断完善。行政监察和审计监督力度加大，信息公开和政务公开制度不断完善，廉政建设和反腐败工作扎实推进。

各位代表，2015年工作任务的完成，标志着“十二五”规划主要目标的顺利实现。过去五年，我们主动适应经济发展新常态，自加压力，真抓实干，攻坚克难，发展实现新的跨越。

五年来，我们坚定不移加快绿色发展，经济增长和生态保护协同共进。抢抓行政区划调整、高铁通车等重大历史机遇，率先提出并坚持实施绿色发展战略，综合实力明显增强。全市生产总值年均增长10.6%，这五年是改革开放以来与全省平均水平差距最小的时期。一般公共预算总收入、地方一般公共预算收入均比2010年翻一番，全社会固定资产投资达到2010年的3倍。深化产业梳理，制定和实施千亿五百亿产值行动计划，加强专业产业招商，“5+3”产业和特色现代农业加快培育。滚动实施“迎高铁抓旅游促三产”行动计划，以旅游为主的第三产业拉动力明显增强。绩效考评连续居全省前列。县域经济实力提升，4个县（市）进入全省十佳县。在加快发展的同时，更加重视生态保护。成功争取国家节能减排、循环经济、低碳城市、智慧城市、水生态文明城市、可持续发展实验区等“国字招牌”。森林覆盖率从71.14%提升到76.46%，努力实现不砍树也能致富。主要流域水质功能达标率100%，空气质量优良天数比例99.8%，提前一年完成“十二五”节能减排任务，生态环境质量保持全国全省前列。

五年来，我们持之以恒提升基础设施，投资环境和城乡面貌全面改善。全市人民期盼已久的合福高铁正式通车，南平全面进入“高铁时代”。铁路营运里程新增203公里、总里程733公里，高速公路通车里程新增510公里、总里程933公里，铁路、高速公路总里程均居全省首位，实现县县通高速、镇镇通干线、村村通客车。武夷山机场迁建场址获批，闽江内河航运前期工作取得突破。市本级和各县（市、区）专业产业园区按照“1+N”模式加快建设、初具规模。持续共建美丽南平，武夷新区建设全面提速，延平新城建设同步推进，每年实施一批“百千万”工程和“六个一”项目、打造一批美丽乡村，城乡面貌显著改观。

五年来，我们先行先试推进机制创新，发展活力和内生动力明显增强。国有企业整合重组成效显现，9家国有集团公司总资产673.4亿元，成功融资215亿元。建立农村卫生保洁长效机制，万人保洁“南平经验”成为全省典型。环境污染第三方治理试点成功，正在全省全面推广。统一打造武夷品牌，创新武夷旅游统一营销机制，先后开展“一元门票游大武夷”、“低碳之旅·畅游武夷”活动，大武夷旅游格局加快构建；统一打造武夷食品营销平台，绿色产品销售渠道进一步拓宽。“南平机制”持续深化，建立健全“三级联动、重心下移”、“六要”群众工作法、“民生110”网格化管理、农村社会治理“1+3”等长效机制。市县政府机构和工商、质监、食品药品监管体制等改革顺利完成，行政审批制度、商事制度改革持续深化，事业单位分类改革稳步推进。

五年来，我们大力发展社会事业，公共服务和民生福祉显著提高。民生支出占一般公共预算支出的比重超过75%，累计完成为民办实事项目151项。城乡居民人均可支配收入年均分别增长10.7%和12.5%，累计新增城镇就业12.37万人、转移农村劳动力27.62万人。10个县（市、区）列入原中央苏区县。提前实现“双高普九”，科技、文化、卫生、体育、人口计生等社会事业全面进步。社会保障政策全面落实，法治建设进一步加强，综治考评连续三年居全省第一，社会更加安定和谐。

各位代表，成绩来之不易。这是省委、省政府和市委正确领导、全市人民奋力拼搏及各方面大力支持的结果。我代表市人民政府，向全市人民，向人大代表、政协委员、各民主党派、工商联、各人民团体、无党派人士，向离退休老同志和社会各界人士，向中央、省直驻延机构和驻军部队、武警官兵、公安民警，向对口帮扶部门和地区，向关心支持南平发展的港澳同胞、台湾同胞、海外侨胞和国际友人，表示衷心的感谢！

在充分肯定成绩的同时，我们也清醒地认识到发展中还存在不少困难和问题。主要是：经济总量小，产业结构不够优，竞争力和自主创新能力偏弱；大项目不多，尤其是生产性大项目比较缺乏；龙头企业偏少、带动力不强，部分企业经营比较困难；脱贫攻坚任务依然较重；城乡建设管理水平还有待提高，防灾减灾、防洪排涝、停车场所、地下管网等设施还比较薄弱；社会保障体系仍不够完善，教育、卫生等社会事业发展相对滞后；政府职能转变还不到位，法治政府建设有待加强。我们必须高度重视这些问题，采取有效措施认真加以解决。

二、实施“十三五”规划，开创经济社会发展新局面

提交大会审议的《南平市国民经济和社会发展第十三个五年规划纲要（草案）》，提出了今后五年经济社会发展的指导思想、目标任务和政策措施，到2020年全市生产总值达到2000亿元，提前两年实现城乡人均收入比2010年翻一番。

“十三五”时期我市经济社会发展的指导思想：高举中国特色社会主义伟大旗帜，全面贯彻党的十八大和十八届三中、四中、五中全会精神，以马克思列宁主义、毛泽东思想、邓小平理论、“三个代表”重要思想、科学发展观为指导，深入贯彻习近平总书记系列重要讲话和对福建工作的重要指示精神，落实“四个全面”战略布局，牢固树立和贯彻创新、协调、绿色、开放、共享的新发展理念，坚持发展第一要务，以保持经济稳定较快增长为目标，以转型升级为主线，以提高发展质量和效益为中心，以发展成果人民共享为根本目的，全面推进经济、政治、文化、社会、生态文明建设和党的建设，积极创建全国绿色发展示范区，加快形成适应和引领新常态的体制机制，确保如期全面建成小康社会，努力建设机制活、产业优、百姓富、生态美的新南平。

（一）坚持绿色发展，增创发展新优势。牢固树立“绿水青山就是金山银山”的理念，进一步把生态优势转化为发展优势，增强区域竞争力。统一打造武夷品牌，把绿色产品卖出去、把外地游客引进来，扩大绿色产品有效供给和消费需求。深入实施水、大气、土壤污染防治计划，加强水土流失重点区域、重点流域水环境综合治理。倡导文明、绿色生活方式和消费模式，营造守护绿色的浓厚氛围，让南平的山更青、水更绿、空气更清新。

（二）坚持创新引领，培育发展新引擎。深入实施创新驱动和人才优先战略，完善科技创新服务平台，加快人才引进和培养，推动大众创业万众创新。全力培育“5+3”产业和特色现代农业，促进工业化与信息化、“互联网+”与“绿色+”深度融合，打造绿色产业升级版。进一步完善铁路、高速公路、轻轨、内河航运、机场等现代立体交通体系，加快水利、能源、环保、信息、防灾减灾等基础设施建设，提升经济社会发展支撑能力。

（三）坚持统筹协调，拓展发展新空间。统筹城乡一体化发展，加快新型城镇化进程。武夷新区新兴中心城市基本形成，实现南平行政中心搬迁。延平新城加快打造“山水宜居之城、活力创业之城”，中心城区功能不断完善。县城辐射带动能力增强，美丽城乡展现新姿。全面打赢脱贫攻坚战，到2018年现行国定扶贫标准贫困人口全面脱贫，2020年现行省定扶贫标准贫困人口全面脱贫、5个省级扶贫开发工作重点县全部摘帽。加强社会主义精神文明建设，弘扬社会主义核心价值观，促进文化事业全面繁荣，加快文化产业发展，推进文化强市建设。

（四）坚持改革开放，激活发展新动力。落实供给侧结构性改革措施，培育新供给新动力。深入推进国资国企、市场准入、投融资、土地流转、林权交易、碳排放交易、流域生态补偿等领域改革，协同推进简政放权、放管结合，优化投资发展环境。主动融入“一带一路”、自贸区建设，深化对台交流合作，密切港澳侨合作，拓展山海协作，加强跨区域协作，形成全方位、多层次的开放交流合作格局。

（五）坚持包容共享，顺应发展新期待。把新增财力更多用在民生事业上，持续办好为民实事，加快补齐民生短板。健全公共就业创业服务体系，推动更高质量就业。坚持优先发展教育，推进优质教育资源更加均衡，办好人民满意的教育。深化医药卫生体制改革，完善医疗卫生服务体系，保障食品药品安全。健全社会保障体系，大力发展社会救助和社会福利。加强和创新社会治理，推进法治南平、平安南平、诚信南平建设。

三、扎实做好2016年工作

今年是“十三五”开局之年。经济社会发展的主要预期目标是：全市生产总值增长9%，力争更快更好些；一般公共预算总收入和地方一般公共预算收入均增长6%，全社会固定资产投资增长18%，外贸出口增长6%，验资口径实际利用外资增长10%，社会消费品零售总额增长12%，居民消费价格总水平涨幅控制在3%左右，城镇登记失业率控制在4.2%以内，人口自然增长率控制在9.5‰以内，城镇居民人均可支配收入增长9%，农村居民人均可支配收入增长9.5%，完成年度节能减排降碳任务。

实现上述目标，重点抓好八个方面工作：

（一）抓牢抓实重点项目，打好扩大投资组合拳

多措并举扩大投资。把投资重点放在调结构、补短板、惠民生上，紧紧围绕“谋划一批、签约一批、开工一批、投产一批、增资一批”，加快实施一批产业、交通、能源、城乡建设、农林水、环保等重大项目。加大“三比一看”力度，坚持“一月一协调、一季一督查”工作机制，建立健全项目策划联席会议、项目定期通报、表格化管理等机制，加强项目协调调度，做好项目审批服务和要素保障，增强项目推进实效。

加快重点项目建设。实施重点项目346个、总投资2419亿元，其中在建项目258个、年度投资350亿元，力争新开工130个、投产87个。建成闽江上游富屯溪四期防洪工程等项目；加快建设衢宁铁路、南三龙铁路、顺邵高速、南平联络线等项目，新改建18个国省道项目，推进新一轮电网改造升级工程；加快推进浦建龙梅铁路、吉武温铁路、鹰厦铁路北段扩能改造、武沙高速、延尤高速、闽江内河航运、武夷山机场迁建等项目前期。

持续加强招商引资。坚持“走出去”、“请进来”，加强专业招商、园区招商和以商招商，强化点对点、小分队招商。围绕全产业链发展，突破技术链、价值链、产业链关键环节，引进一批龙头企业和上游关键材料、核心部件和下游终端产品项目。完善“5·13”招商项目推进机制，办好“11·16”茶博会，力争投资额2000万元以上项目开工260个以上，增强经济发展后劲。

（二）创新提升旅游发展，增强第三产业拉动力

推动旅游产业提档升级。充分发挥“双世遗”品牌优势，持续开展“低碳之旅·畅游武夷”活动，力争旅游接待人数和收入分别增长16%和18%。加强资源要素整合和精品线路策划，加快推进航天主题武夷体验园、武夷山五夫朱子文化园、建阳考亭旅游文化园、政和佛子山旅游区、邵武金坑红色旅游区、顺昌宝山和合掌岩旅游区等项目，力争新增国家3A级以上旅游景区7家。加强多元化业态培育，推动旅游与一二三产深度融合发展。加快住宿、餐饮、景区景点、旅行社等行业标准化建设，推广智慧旅游，打造智能化、人性化的旅游服务平台。

培育激活新型业态。统一打响武夷区域品牌，引导名特优新产品生产企业联手开拓市场，扩大武夷产品的国内外知名度。统一打造绿色产品营销平台，鼓励圣农、太阳电缆等龙头企业牵头组建营销联盟，创新产品营销模式。大力发展电子商务，打造武夷旅游网“513购”等电商平台，发展农村淘宝，培育壮大一批电商孵化基地、集聚园区和龙头企业，实现线上线下融合发展。

大力发展现代服务业。加快现代物流业发展，规划建设一批大型综合物流园区和冷链物流项目。加快建设城市商贸综合体、特色商业街、农产品批发市场和城乡集贸市场。推动批发零售、住宿餐饮、市政服务、农村服务等传统服务业改造升级，发展健康养老、文体娱乐、休闲养生、家政服务等新兴产业。支持金融业发展，继续筹建一批农商银行、村镇银行，引进股份制商业银行，完善金融服务体系。有效防范和化解金融风险，规范民间融资行为，守住不发生系统性和区域性金融风险的底线。

（三）做大做强工业经济，打造绿色制造业升级版

培育壮大优势产业。加快产业转型升级，注重从供给侧发力，提升存量、做大增量，推动传统优势产业集聚化发展、新兴产业规模化发展。加快实施66个重点工业项目、30个市级智能制造重点项目，提升食品加工、机电制造、竹加工、新型轻纺等产业技术装备水平，延伸产业链条，提高产品附加值。实施新兴产业倍增计划，大力培育生物医药、新能源新材料、节能环保等产业。加快专业园区配套设施建设，鼓励老企业退城入园。

提升创业创新活力。健全以市场为导向、以企业为主体的产学研用机制，引导科研院所、高等院校面向企业开展技术创新。加快省级以上工程技术中心、院士工作站、知识产权服务工作站等创新平台建设，打造一批创业创新示范基地和新型众创空间。争创国家级小微企业创业创新示范基地城市。探索“孵化器+创业风险投资”模式，引进和培育创业投资、风险投资，鼓励支持科技人员领办、参办科技型小微企业。

支持企业加快发展。持续开展送政策进企业活动，全面落实降低企业成本的政策措施。实施产业龙头促进计划，支持龙头企业增资扩产、延伸链条、兼并重组，新培育产值超10亿元企业10家。实施中小微企业成长计划，完善产业分工协作体系，新增规模以上工业企业70家。鼓励使用地产名优工业品，帮助企业开拓市场。加强政银企对接，创新金融服务方式，拓宽企业融资渠道，力争10家企业在主板或“新三板”挂牌。新申报福建名牌产品15项，新增中国驰名商标2件、省著名商标30件。

（四）做特做优现代农业，全力打好脱贫攻坚战

加快发展特色优势农业。稳定粮食面积，提高粮食产能。围绕打造全国绿色农产品基地，做大闽北特色优势农产品规模。建立健全土地流转、金融、科技、人才、营销、品牌等服务平台，培育农业产业化龙头企业、农村合作组织和种养大户。加快发展农副产品精深加工、乡村旅游和农村电子商务，推动一产接二连三，带动农民增收致富。加快现代农业示范区、农民创业园、农业科技园和农民创业示范基地建设，支持智能温控大棚、无土基质栽培等设施农业发展，培育25个林下经济示范基地。落实竹林生态保护开发机制，完成竹山机耕路1000公里、灌溉面积1万亩、施肥1万亩，优化竹产业布局。推进“三品一标”认证，严格农产品质量安全监管，加强重大动植物疫病防控。做好第三次全国农业普查前期准备工作。

提升农业综合生产能力。加强农业“五新”推广，加快省农科院闽北分院建设，推动农业科技、种业创新，提升农业机械化水平。重点实施5座水库工程、10段防洪工程、7条流域综合治理工程，继续推进小型农田水利重点县建设，实施农村饮水安全巩固提升工程。新改建农村公路200公里，实施撤渡建桥及危桥改造30座，新建农村客运候车亭50个，改善农村生产生活条件。

构建新型农业经营体系。坚持和完善农村基本经营制度，探索开展农村土地承包经营权、农民住房财产权“两权”抵押贷款试点。加快发展家庭农场，引导农民合作社规范化建设，积极推进4个新型职业农民培育试点县工作。发展智慧农业、定制农业等新型业态，实现农业生产精准化种植、可视化管理。

实施脱贫攻坚工程。聚焦精准要求，健全“市县抓落实、工作到村、帮扶到户”的长效机制，充分发挥乡镇作用，构建全社会协同推进的大扶贫格局，全年实现脱贫3万人。突出精准识别，坚持贫困标准，坚持公开公平，规范建档立卡，确保扶贫对象到户到人。突出精准施策，落实挂钩帮扶政策，深化山海协作，分类制定帮扶措施，实行发展产业脱贫、转移就业脱贫、“造福工程”搬迁脱贫、发展教育脱贫、生态补偿脱贫、低保兜底脱贫、医疗保险和医疗救助脱贫，提高脱贫实效。突出精准管理，建立贫困户脱贫退出认定机制，对已经脱贫的农户加强跟踪服务，在一定时间内继续享受扶贫政策，确保扶真贫、真扶贫、真脱贫。

（五）加快建设武夷新区，积极推进新型城镇化

举全市之力建设武夷新区。围绕尽快实现行政中心搬迁目标，集中力量、能快则快建设武夷新区。坚持把以人为本、尊重自然、传承历史、绿色低碳等理念融入新区规划建设的全过程，彰显绿色风貌和闽北特色。全力推进南林核心区建设，加快建设云谷小区、歌剧院、艺术中心、文化馆、图书馆、规划展示馆、崇阳溪综合治理、快速通道、轨道交通、市政工程；开工建设水资源配置工程、绕城高速、森林生态博物院、市民公园、客运总站、南林核心区商务集中区等项目，力争全年完成投资210亿元以上、增长50%。争取申报列入国家海绵城市、综合管廊建设试点。提升新区产业支撑能力，加快高新技术产业园区建设，引进和培育各类企业总部、行业总部，加快发展食品加工、旅游养生、机械电子、生物医药和新能源新材料产业。推进新区与建阳、武夷山同城化发展，促进城市建筑风格相互协调、产业空间布局相互衔接、交通基础设施互联互通。

加快延平新城建设。推进316国道炉下至东坑段改扩建及沿线产业带开发和南平港延平新城港区码头、闽江源临港物流园建设，加快延平新城从彦路、海瑞路、水鸠路、成功路、工业一路、工业二路、陈瓦路等道路建设和道路绿化工程、污水管网一期工程、江南片区水厂及管网工程建设；完善陈坑-瓦口产业组团配套设施，推进增坑食品加工专业园建设，促进产城融合发展。加快西芹大桥、莲花山大桥、社区体育公园、中小学校、东坑岭铁路棚户区改造等项目建设，促进新老城区联动发展。

提升城市综合承载能力。遵循城市发展规律，加强城市规划建设管理。完成南平“一市两区”总体规划编制，完善各县（市、区）控制性详细规划。加快国家级、省级新型城镇化试点、小城镇综合改革试点建设，不断提升城镇化质量。持续实施“六个一”项目，把城市文化特色区、城市公园、商贸街区等“串联”起来，展示城市特有的环境特质、文化特色和建筑风貌。实施宜居环境建设行动计划、新一轮市政提升“五千工程”，抓好城市道路、公共停车场、休闲慢道等建设。推动建筑业转型升级和房地产业持续健康发展。创新城市治理方式，深化城市执法体制改革，加大“两违”整治力度。加快户籍制度改革，促进农业转移人口融入城镇，提高户籍人口城镇化率，推进基本公共服务均等化。

持续建设美丽乡村。深入实施“百千万”工程，重点推进中心村镇以及重要通道和重要流域沿线、重要区域周边的村庄整治建设，打造美丽乡村景观带。加强历史文化名镇名村、历史文化街区、历史建筑、传统村落保护，留住民俗风情，突出武夷风格建筑风貌，建设一批各具特色的美丽村庄。创新村庄建设和治理模式，建立村民共建共管长效机制。

（六）持续保护绿水青山，建设生态文明先行示范区

深入推进节能减排。严格落实环保监管“一岗双责”，实行能源和水资源能耗、建设用地等总量和强度双控。落实节能减排责任制，加快节能减排示范项目建设。开展产业园区循环化改造，实施重点用能企业能耗在线监测和管理，推进工业园区污水集中治理、化工类重点行业污染深度治理。开展排污权交易工作，全面完成试点行业初始排污权核定。推广节水技术改造和居民生活节水工作。探索开展低碳产品标准制定和低碳产品认定工作。

强化生态环境保护。持续推进生态创建工作，争取4个县（市）获得国家生态县命名。加强生态茶园建设，植树造林18.95万亩，加快环城一重山绿化美化，积极创建省级森林城市。落实“河长制”，深化闽江流域水环境综合整治，完成水土流失治理25万亩，建设100公里安全生态水系工程，启动4个县城区应急备用水源建设，加快一批污水处理设施建设。推进清洁生产，加大黄标车淘汰力度，改善空气质量。加强城乡生活垃圾压缩转运站、转运系统及行政村收运设施建设，开展打击违法排污企业保障群众健康环保专项行动，加强涉重金属行业污染防治。

健全生态文明制度。开展领导干部自然资源资产离任审计试点。完善国土空间开发保护机制，科学合理划定生态功能红线，稳妥推进武夷山国家公园体制试点。实施山水林田湖生态保护和修复工程，加强土地矿产执法监察，限制新矿产勘查开发行为。落实生态补偿制度，健全生态公益林发展机制，扩大重点区位商品林收储试点范围。全面推行环境污染第三方治理，建立政府、企业、公众共治的环境治理体系。

（七）深化改革扩大开放，激发经济社会发展活力

深化体制机制创新。深入推进行政审批制度改革，全面推行“三张清单”，进一步取消和下放审批事项。全面落实行政审批“三集中、三到位”改革，建立完善市级中介服务中心。开展武夷新区投资项目审批制度改革试点，完善工程标准化建设管理和项目运作机制。简化企业投资审批程序，分类分期减少企业投资项目前置审批。完善国有资产监督管理体制，加强国有企业结构调整与重组，健全公司法人治理机构。持续推进商事制度改革，全面实行“一照一码”和电子执照制度。继续深化财税体制改革，推进预算绩效管理，改进财政资金支持方式，推动政府与社会资本合作，扩大政府购买服务范围，撬动更多社会资本。

拓展对外交流合作。主动融入“海丝”核心区和福建自贸区，发挥武夷山“万里茶道”海陆双起点的独特优势，深化与沿线城市合作。拓展对台合作领域，推动向更广范围、更高层次迈进。优化外贸通关环境，推进关检合作“三个一”模式，加快武夷山“陆地港”发展，打造内陆地区口岸的重要支点和集疏运通道。完善促进外贸发展政策，支持企业参加国内外产品展示展销会、订货会，发展跨境电商，推动出口市场多元化，扩大出口需求。

（八）保障和改善民生，让群众拥有更多获得感

织好就业和社会保障网。实施更加积极的就业政策，鼓励以创业带就业，统筹抓好高校毕业生、就业困难人员、农村转移劳动力等群体就业，新增城镇就业1.8万人。实施全民参保计划，深化机关事业单位养老保险制度改革，推进养老、医疗保险从制度全覆盖向人员全覆盖，完善社会保险关系转移接续办法。全面推行复合型医保支付方式，强化医保基金收支预算。健全完善企业欠薪应急保障金制度。加大棚户区改造力度，落实保障性安居工程8425套（户）。全面实施临时救助制度。继续开展农村幸福院项目建设，完善农村养老服务设施。健全农村留守儿童、妇女、老年人、失独家庭关爱服务体系，积极发展慈善和残疾人事业。

全面发展社会事业。认真办好为民办实事项目。统筹城乡义务教育资源均衡配置，加快推进薄弱校改善工程和城区优质校扩容工程。继续实施第二轮学前教育三年行动计划，新扩建公办幼儿园10所。开展特殊教育提升计划，重视发展终身教育和老年教育。加快发展职业教育，整合职业教育资源，深化产教融合和校企合作。支持武夷学院向应用技术型大学转型。实施名师名校培养工程，加强教师队伍建设。持续实施“5115”人才集聚工程，创建一批人才工作联系点。深化医药卫生体制改革，进一步推进公立医院和基层医疗卫生机构综合改革，加快武夷总医院发展，组建医疗联合体，推进分级诊疗制度建设。鼓励社会力量办医，加强全科医生和乡村医生队伍建设，提高基层医疗服务水平。全面实施两孩政策，促进人口长期均衡发展。推动群众体育和竞技体育全面发展，培育体育产业。推进公共文化服务体系建设，加强文化遗产保护传承与利用，创建朱子故里海峡两岸交流基地，打响朱子文化等特色品牌。推进新型智库建设。办好新闻出版广播影视事业。做好档案和二轮修志工作。

提升社会治理能力。完善重大决策社会稳定风险评估机制，坚持领导干部接访下访机制，严格实施信访“路线图”，健全完善“大调解”工作体系。总结提升医患纠纷调处“南平解法”等做法，完善多元化矛盾纠纷调解机制。深化“平安南平”创建，创新“互联网+社会服务”，健全完善社会治理网格化服务管理体系和立体化社会治安防控体系，加强反恐怖工作。全面落实企业安全生产主体责任，加强安全生产监管执法能力建设，推进安全生产标准化建设，深入开展消防隐患排查整治，持续深化道路交通安全综合整治。启动实施“七五”普法，完善公共法律服务体系。加强社区网格化服务管理，健全社区矫正制度。完善食品药品安全监管机制，加强检验检测机构能力建设。支持工会、共青团、妇联等群团组织积极开展工作。做好民族宗教工作。抓好新一轮全国“双拥模范城”创建工作，支持驻军部队和武警部队建设，强化国防教育、国防动员、国防后备力量和人民防空建设。

四、加强政府自身建设，全面提升执政能力

忠心忠诚，践行宗旨。自觉践行“三严三实”，坚定理想信念，严守政治纪律和政治规矩，强化看齐意识，始终在思想上政治上行动上与党中央保持高度一致，不折不扣贯彻落实省委省政府和市委的决策部署。树牢全心全意为人民服务的根本宗旨，解决好涉及群众切身利益的问题，让改革发展成果更多惠及人民。

敢闯敢试，勇于担当。进一步解放思想，以五大发展理念引领新发展。大力倡导实干拼创，保持强烈的责任心和使命感，不搞短期行为，不做表面文章，对看准的事紧抓不放、迎难而上，坚定不移地向前推进。持续深化简政放权，加快转变职能，创新政府管理。完善创业竞赛和绩效考评办法，激励干部主动作为、大胆作为，营造良好的干事创业氛围。

从严从实，改进作风。进一步深化机关效能建设，精简文件、压缩会议，马上就办、办就办好，坚决纠正“庸懒散拖”，不断提高执行力。进一步深化“南平机制”，持续完善“三级联动、重心下移”、“六要”群众工作法、“民生110”网格化服务等做法，形成更加直接、便捷、高效的联系服务群众机制。

干净干事，廉洁从政。严格落实党风廉政建设责任制，深入贯彻中央“八项规定”精神，认真执行《中国共产党廉洁自律准则》、《中国共产党纪律处分条例》，把廉洁从政贯穿到政府工作的各个环节。严格用制度管权管事管财管人，抓好土地出让、工程建设、产权交易、政府采购等重点领域廉政风险防控。自觉接受人大、政协监督，接受司法、舆论和群众监督，加强行政监察和审计监督，确保权力廉洁高效运行。

各位代表，开创新局面、建设新南平的目标催人奋进。让我们紧密团结在以习近平同志为总书记的党中央周围，在省委、省政府和市委的领导下，凝心聚力，开拓进取，扎实工作，为全面建成小康社会、实现科学发展跨越发展作出新的更大的贡献！
